# Annotations

**Published:** 1893-07-01

**Authors:** 


### ANNOTATIONS.

**Neolithic Surgery.**

Of all the professions and arts medicine acknowledges the smallest obligations to the past. The average medical man is convinced that we moderns are the men, and that wisdom, if it will not die with us, was at any rate born with us. This is not at all a correct view of medicine as an art, as, indeed, a moment's reflection may convince the dullest. There probably never was a man anywhere who did not try some method of relief when in pain, some attempt at cure when injured and disabled. Even the dog will lick his own wounds, and the cow, like a careful midwife, licks clean her newly-born calf. The *Spectator* in an interesting review of the Marquis de Nadaillac's "Manners and Monuments of Prehistoric Peoples," reminds us that surgery had attained a considerable boldness and success even so far back as the Neolithic period. "In several instances we can make out fractures set with a neatness which gives us a very high opinion of the skill of the Neolithic bone-setters. There are evidences of more difficult operations than bone-setting. Many examples of trepanning have been found in which the conditions of the edges of the orifice show that the patient recovered. In a skull found under the Lozère Dolmen a piece of bone had been let into the parietal bone as large as a five-franc coin. In some cases the operation seems to have been performed for medical reasons—to relieve epilepsy or other cerebral affections. Most of the examples of trepanning appear to belong to Neolithic or later times, but there is some evidence to show that the operation was performed during the Paleolithic age, and with no other instrument than a sharp stone." Possibly there is not much of an immediately practical kind to gain by the study of venerable medical monuments; but there is at least a sense of proportion to be acquired, and a capacity for doing justice to predecessors who were much more worthy of honour than we are at all inclined to allow. We English probably know less of medical history than any other civilised nation. What a pity it is that every medical school is without a well-endowed chair of medical philosophy and history. Great physicians and surgeons have seldom been benefactors to medical learning. Perhaps some of our living physicians who experience difficulty in finding investments for their accumulating guineas will think of this subject and endow two or three professorships at their universities.

**"I decline to meet you."**

It is rightly considered an unpardonable insult for one medical man to refuse to meet another in consultation without reasons of the most adequate and convincing character. A month or two ago, a practitioner in a country town called upon another practitioner in the same town, and asked him to see a patient in consultation with himself. "Are not you Dr. —— who keeps a sixpenny dispensary?" asked the consultant, who, by the way, was a general practitioner. "I am!" "Then I decline to meet you!" Was this refusal to meet another member of the same profession justifiable and right? and, if so, on what ground was it justifiable and right? We are of opinion that it was entirely right; and that the doctor who declined the consultation rendered a service to medicine which was worthy of general imitation. The first practitioner kept a sixpenny dispensary; and for that reason the second declined to meet him in consultation. But the unreflecting may ask, has not a man a right to charge any fee he pleases for his services; has he not a right to see a patient without charging a fee at all? Undoubtedly he has! But to charge an inadequate fee occasionally and for special reasons is one thing; to charge it systematically, and as a matter of regular business is quite another, and opens up a different class of questions altogether. It leads, in fact, directly and inevitably to fraud. A man makes it known that he will give advice and medicine to anyone who chooses to accept it, that is practically to everyone, for sixpence. But no man can give competent advice and serviceable medicine to all and sundry for sixpence each. It is impossible. To profess to do so is the same as professing to sell sovereigns for five shillings each. If any man were to sell sovereigns on a large scale for five shillings each, and were to make a living by the transaction, the stupidest person in the world would know that the sovereigns must be counterfeits, and that the seller must be a cheat. It is exactly the same with the sixpenny dispensary business. It follows that the sixpenny dispensary can only be carried on profitably by being carried on dishonestly. Every medical man knows this. The consultant who meets the sixpenny dispenser knows that he is encouraging fraud. Whether is more honourable, to be polite and encourage cheating at the bedside of the sick and the dying, or by declining to meet a sixpenny dispenser to make a stern and necessary protest against one of the most cruel and dangerous frauds which can be practised upon a human being? There can only be one reasonable answer to this question.

**Hip Disease in Children.**

Family practitioners can hardly be specialists in every branch of operative medicine and surgery; but they ought to aim at being more or less of specialists in the symptomatic department of every common disease. In other words, they ought to know well what they may properly deal with themselves, and quite as well what they ought at once to pass on to the operative specialist. The business of every medical man, be he specialist, or be he general practitioner, is either to do himself, or to get done, for his patient the very best of which the most advanced modern science is capable. An article in the *Practitioner* for June, by Mr. Howard Marsh, F.R.C.S., well illustrates the advantages of competent early watchfulness in so frequent, dangerous, and maiming an affliction as hip-joint disease; and shows also what misery and helplessness may be prevented by intelligent parents. One brief narrative teaches us by an example how possible it is for the most competent surgeons to make mistakes, and how such mistakes may be minimised by parents who are critical and courageous. "A highly distinguished surgeon," Mr. Howard Marsh tells us, "expressed the opinion that a certain boy of nine had early hip disease, and advised that he should be at once confined to the horizontal position. After the boy had been kept in bed a fortnight, his parents decided to see some one else, and consulted a surgeon scarcely less distinguished than the first. This surgeon said that no disease was present. Feeling all at sea, the parents requested me to examine the child, but without stating what had already taken place. . . . . I reported, after examination, that a suspicion of hip disease must be entertained. . . . The view that hip disease was present was confirmed by the subsequent history of the case." Mr. Howard Marsh does not tell us the final issue; but we may readily understand that in many such cases early diagnosis prevents untold weariness and suffering, and saves the patient from the maiming and disfigurement of a shortened limb; whilst in not a few instances it is the "stitch in time" which saves life. Such a story confirms our oft repeated contention, that one of the prime duties of medical leadership is to raise the level of general knowledge, culture, and capacity among the rank and file of the profession. It is only in this way that the advancing science of medicine can render to modern peoples the truly wonderful services which it is capable of rendering.

